# Balloon-Assisted Maturation in Salvaging Stenosed Arteriovenous Fistulas: A Case Series and Clinical Insights

**DOI:** 10.7759/cureus.114107

**Published:** 2026-08-07

**Authors:** Sumeet David, Kailash Chander, Angel, Damanbir S Chahal, Neena Bhatti, Girish Joseph

**Affiliations:** 1 Cardiology, Lifeline Heart Centre, Jalandhar, IND; 2 Anatomy, Jaipur National University Institute for Medical Sciences & Research Centre, Jaipur, IND; 3 Urology, Lifeline Heart Centre, Jalandhar, IND; 4 Pharmacology and Therapeutics, Christian Medical College & Hospital, Ludhiana, IND

**Keywords:** arteriovenous fistula, balloon-assisted maturation, end-stage renal disease, fistula maturation failure, hemodialysis vascular access, percutaneous transluminal angioplasty

## Abstract

Background

An autogenous arteriovenous fistula (AVF), created by direct anastomosis of an adjacent artery and vein, is the preferred vascular access for maintenance hemodialysis in patients with end-stage renal disease (ESRD). Failure to mature is nevertheless common, and a substantial proportion of newly created fistulas require an adjunctive procedure before they can be cannulated. Balloon-assisted maturation (BAM) is the most widely used endovascular technique for salvaging such fistulas.

Methodology

This descriptive case series was conducted at Kidney Hospital and Lifeline Medical Institutions, Jalandhar, India, between July 2024 and December 2024. Consecutive patients undergoing first-time autogenous AVF creation were reviewed at four and eight weeks with clinical examination and Doppler ultrasound. Patients whose fistulas were judged immature were offered BAM. In total, 10 patients underwent the procedure: eight radiocephalic and two brachiocephalic fistulas. Angioplasty was performed under local anesthesia and fluoroscopic guidance through the outflow vein, with the stenosis crossed using a hydrophilic guidewire and dilated sequentially. Written informed consent was obtained from every patient.

Results

Of the 84 patients undergoing first-time autogenous AVF creation during the study period, 18 (21.4%) had an immature fistula with a demonstrable stenosis at follow-up; eight were managed conservatively, and 10 underwent BAM. The mean age of the BAM cohort was 60.7 years (range = 46-73), and eight (80%) patients were male. Diabetes mellitus and hypertension were present in all 10 patients, and diabetes was the cause of ESRD in eight. Technical success was achieved in all 10 (100%) fistulas. The majority of culprit lesions were located within the juxta-anastomotic venous segment. Completion fistulograms showed a smooth luminal contour, increased venous calibre, and no contrast extravasation along both the treated segment and the entire usable length of the fistula. There were no venous ruptures, access-site hematomas, or post-procedural thromboses, and all 10 fistulas were subsequently cannulated with two needles for hemodialysis.

Conclusions

In this small series, BAM was a safe and effective adjunct for salvaging stenosed, non-maturing AVFs, with a 100% technical and clinical success rate and no procedural complications. The findings are consistent with larger published cohorts, but the absence of a comparator group, the small sample, and the short follow-up preclude any conclusion about long-term patency; larger prospective studies with defined patency endpoints are required.

## Introduction

An autogenous arteriovenous fistula (AVF), formed by the direct anastomosis of an adjacent artery and vein, is recommended as the first-choice vascular access for patients with end-stage renal disease (ESRD) who require maintenance hemodialysis. Compared with arteriovenous grafts and central venous catheters, autogenous fistulas are associated with lower rates of infection and thrombosis, fewer access-related hospital admissions, and lower long-term healthcare costs, and current Kidney Disease Outcomes Quality Initiative (KDOQI) guidance places fistula creation at the center of an individualized end-stage kidney disease “Life-Plan” [1].

The creation of a fistula bypasses the resistance vessels of the distal extremity and establishes a parallel, low-resistance return pathway to the heart. Adequate perfusion of the tissue distal to the anastomosis depends on the proximal arterial system dilating sufficiently to compensate for the blood shunted through the fistula. When this compensation is inadequate, distal ischemia or dialysis access steal syndrome may develop, a complication reported in approximately 4-10% of patients after access creation and one that is more frequent in brachial-based fistulas and in patients with diabetes, hypertension, and coronary or cerebrovascular disease [2].

A fistula is considered mature when it can be reliably cannulated with two needles and can deliver the blood flow required for the whole dialysis session. The KDOQI “rule of 6s” provides a widely used clinical benchmark: a diameter of at least 6 mm, a depth of no more than 6 mm from the skin, a flow of at least 600 mL/minute, and a usable straight segment of at least 6 cm, assessed approximately six weeks after creation [1].

Despite meticulous surgical technique, failure to mature remains the principal limitation of the autogenous fistula. Preoperative arterial and venous diameters, systemic inflammatory burden, and nutritional status have all been shown to predict maturation failure [3]. The underlying biology is one of maladaptive outward remodelling: aggressive venous neointimal hyperplasia, driven by endothelial injury, inflammatory signalling and oxidative stress, narrows the juxta-anastomotic segment and prevents the vein from dilating in response to the increased shear stress that follows anastomosis [4].

Consequently, a considerable proportion of fistulas require an intervention before they can be used. In a large US cohort, roughly one in four patients required a procedure during the maturation phase alone [5], and prospective trial data indicate that patients with radiocephalic fistulas undergo an average of about one intervention in the first postoperative year, with balloon angioplasty accounting for more than half of all procedures performed [6].

Balloon-assisted maturation (BAM) has emerged as the most commonly used technique for promoting the maturation of such fistulas. Rather than treating a single focal stenosis in isolation, BAM combines angioplasty, healing, and progressive remodelling of the whole fistula into a sequential process, and can also be used to upgrade small-caliber veins that would previously have been considered unsuitable for access creation. As failure of AVF maturation often necessitates repeated imaging, additional endovascular or surgical procedures, prolonged catheter dependence, and delayed establishment of definitive dialysis access, it increases healthcare resource utilization and the overall burden of vascular access care [5]. A systematic review and meta-analysis of 13 studies including 1,427 patients with non-matured fistulas reported a pooled technical success rate of 97% and a pooled clinical success rate of 90%, with manageable complications [7].

Although experience with BAM from India remains limited, a recent retrospective study from Tamil Nadu reported favorable outcomes following endovascular balloon angioplasty for failed hemodialysis AVFs [8]. However, published Indian literature specifically evaluating BAM for stenosed, non-maturing autogenous AVFs remains scarce. Therefore, the primary objective of this study was to evaluate the technical feasibility, clinical success, and immediate procedural outcomes of BAM in consecutive patients with stenosed, non-maturing autogenous AVFs treated at a tertiary care center. We also sought to compare our findings with the contemporary published literature.

## Materials and methods

Study design and setting

This was a descriptive, single-center case series conducted at Kidney Hospital and Lifeline Medical Institutions, Jalandhar, Punjab, India. The study period extended from July 2024 to December 2024. All fistulas were created at our institution, and all were first-time hemodialysis access procedures.

Participants

All consecutive eligible patients meeting the inclusion criteria during the study period were included without selective exclusion. The inclusion and exclusion criteria are summarized in Table 1.

**Table 1:** Inclusion and exclusion criteria for patient selection. ESRD = end-stage renal disease; AVF = arteriovenous fistula; BAM = balloon-assisted maturation

Inclusion criteria	Exclusion criteria
Patients with ESRD undergoing first-time autogenous AVF creation	Previous AVF or arteriovenous graft creation
Clinical and Doppler ultrasound evidence of a non-maturing AVF at four- or eight-week follow-up	Absence of a demonstrable stenotic lesion amenable to endovascular treatment
Angiographically confirmed stenotic lesion considered suitable for BAM	Patients managed without BAM because of progressive spontaneous maturation or mild hemodynamically insignificant stenosis
Written informed consent for the procedure and publication of anonymized clinical data and radiological images	Patients who declined consent

All patients were instructed to return to the outpatient clinic at four and eight weeks after surgery for assessment of maturation. Assessment comprised physical examination (palpation of thrill, assessment of pulse augmentation, and the arm-elevation test) and Doppler ultrasound.

**Figure 1:**
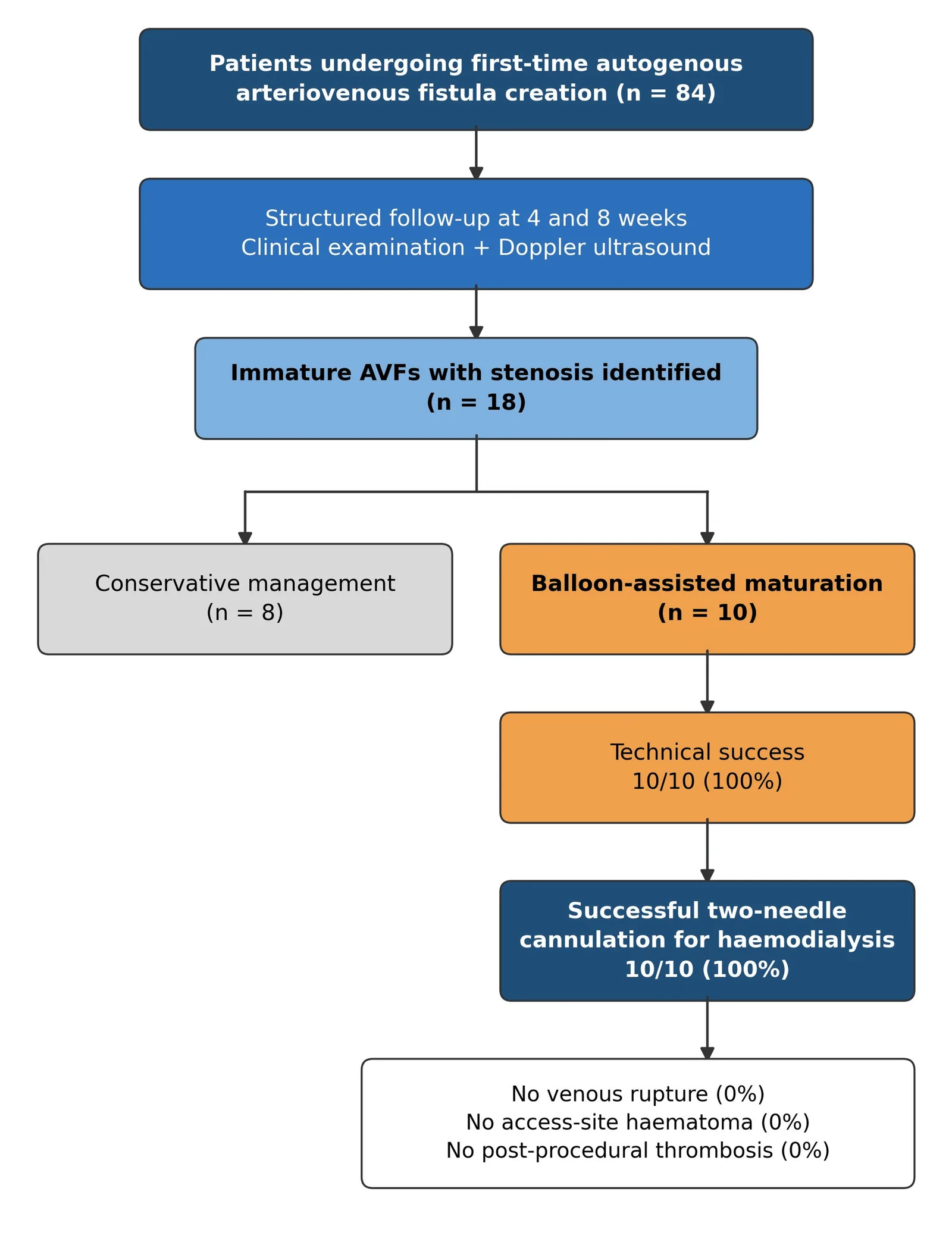
Flow of patients through screening, maturation assessment, and treatment. AVF = arteriovenous fistula; BAM = balloon-assisted maturation

Of the 84 patients who underwent first-time autogenous AVF creation during the study period, 18 (21.4%) were found to have an immature fistula with a demonstrable stenotic lesion at the four- or eight-week assessment. Of these, eight fistulas were managed conservatively with continued surveillance, and the remaining 10 fistulas, in which the stenosis was considered anatomically amenable to endovascular correction, were offered BAM. These patients had either mild stenosis without significant hemodynamic compromise or demonstrated progressive maturation on serial clinical and Doppler assessment, and therefore did not require immediate endovascular intervention. The 10 patients underwent the procedure during the study period and constitute the present series. The overall flow of patients through screening, assessment, and treatment is summarized in Figure 1.

Procedure

BAM was performed under a standardized institutional protocol using local infiltration anesthesia and fluoroscopic guidance. Vascular access was obtained by antegrade puncture of the outflow (cephalic) vein, followed by diagnostic fistulography to delineate the arteriovenous anastomosis, juxta-anastomotic segment, outflow vein, and central veins. Angiographic assessment was performed to identify the culprit stenosis and characterize the lesion with respect to its anatomical location, length, and severity.

The stenotic lesion was crossed using a hydrophilic 0.035-inch guidewire. Pre-dilatation was performed at the juxta-anastomotic venous segment, followed by sequential balloon angioplasty of the stenotic and hypoplastic venous segments. Balloon diameter was selected according to the caliber of the adjacent non-stenotic reference vein and progressively increased in a stepwise manner until satisfactory angiographic flow and luminal expansion were achieved. Balloon size and inflation pressure were individualized according to vessel caliber, lesion morphology, and operator judgement during the procedure, consistent with contemporary BAM techniques that advocate gradual vessel dilatation while minimizing barotrauma and vessel injury. A completion angiogram was obtained in every case to confirm restoration of satisfactory angiographic flow, improvement in luminal caliber, and the absence of significant residual stenosis or contrast extravasation.

Patients were observed after the procedure for hemostasis and access-site complications before discharge. Clinical follow-up was subsequently undertaken to assess successful functional maturation and suitability of the fistula for hemodialysis cannulation.

Outcomes and definitions

Technical success was defined as successful crossing and dilatation of the target lesion with restoration of satisfactory angiographic flow, improvement in luminal caliber, and absence of significant residual stenosis or contrast extravasation on the completion angiogram, as assessed by the operating interventional cardiologist. Clinical success was defined as subsequent cannulation of the fistula with two needles delivering the prescribed dialysis for a complete session. Complications recorded included venous rupture, contrast extravasation, access-site hematoma, and post-procedural fistula thrombosis.

Statistical analysis

Owing to the descriptive nature of this case series and the limited sample size, only descriptive statistical analysis was performed. Continuous variables are presented as mean with range, while categorical variables are expressed as frequency and percentage. No formal hypothesis testing or comparative statistical analyses were undertaken.

Ethical considerations

This report describes a consecutive retrospective case series of patients who underwent BAM as part of routine clinical care. No experimental intervention, randomization, or additional investigations were performed for research purposes. According to institutional policy, formal Institutional Ethics Committee (IEC) approval was not required because this study was a retrospective case series based on patients who had undergone an established procedure as part of routine clinical care. Written informed consent had been obtained from all patients before the procedure as part of routine clinical practice. In addition, written consent was obtained for the use of anonymized clinical data and radiological images. The study was conducted in accordance with the ethical principles of the Declaration of Helsinki.

## Results

**Figure 2:**
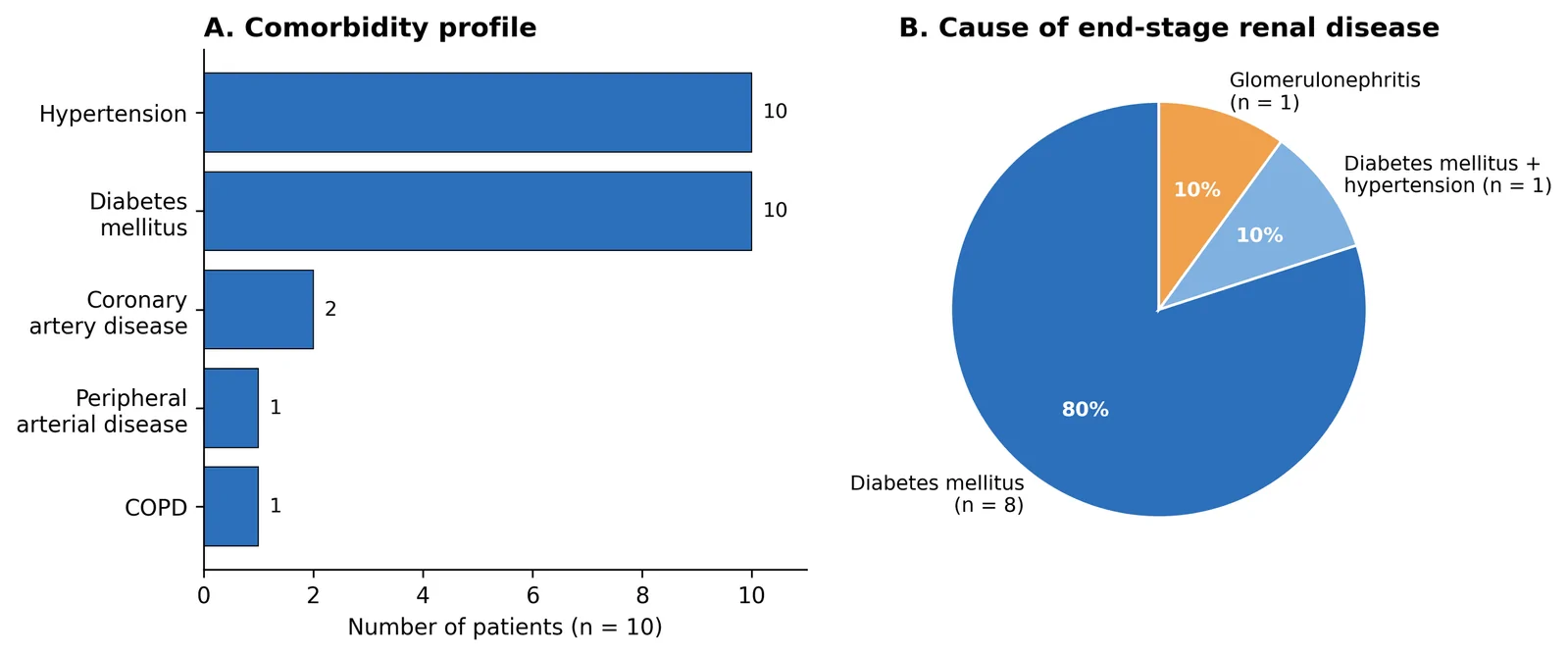
Baseline clinical characteristics of the study cohort. (A) Comorbidity profile of the 10 patients undergoing balloon-assisted maturation. (B) Distribution of the underlying causes of end-stage renal disease. Hypertension and diabetes mellitus were present in all 10 patients. COPD = chronic obstructive pulmonary disease

A total of patients underwent BAM for a stenosed, non-maturing AVF during the study period, selected from 18 immature fistulas identified among 84 patients screened after first-time AVF creation (Figure 1). The mean age was 60.7 years (range = 46-73 years), and eight (80%) patients were male. Eight (80%) fistulas were radio-cephalic, and two (20%) were brachiocephalic. Hypertension and diabetes mellitus were present in all 10 patients; coronary artery disease was present in two, and peripheral arterial disease and chronic obstructive pulmonary disease in one patient each. Diabetes mellitus was the cause of ESRD in eight patients, diabetes with hypertension in one, and glomerulonephritis in one. Baseline characteristics are set out in Table 2, and the comorbidity and etiology profile is summarized graphically in Figure 2.

**Table 2:** Baseline demographic and clinical characteristics of the 10 patients undergoing balloon-assisted maturation. HTN = hypertension; DM = diabetes mellitus; CAD = coronary artery disease; PAD = peripheral arterial disease; COPD = chronic obstructive pulmonary disease; ESRD = end-stage renal disease

Case	Age (years)/Sex	Fistula site	HTN	DM	CAD	PAD	COPD	Cause of ESRD
1	57/Male	Radiocephalic	Yes	Yes	No	No	No	Diabetes mellitus
2	46/Male	Radiocephalic	Yes	Yes	No	No	No	Diabetes mellitus
3	73/Female	Radiocephalic	Yes	Yes	Yes	No	No	Diabetes mellitus, hypertension
4	54/Male	Radiocephalic	Yes	Yes	No	No	Yes	Diabetes mellitus
5	63/Male	Brachiocephalic	Yes	Yes	No	No	No	Diabetes mellitus
6	73/Female	Radiocephalic	Yes	Yes	No	Yes	No	Diabetes mellitus
7	55/Male	Radiocephalic	Yes	Yes	No	No	No	Glomerulonephritis
8	60/Male	Radiocephalic	Yes	Yes	No	No	No	Diabetes mellitus
9	56/Male	Brachiocephalic	Yes	Yes	No	No	No	Diabetes mellitus
10	70/Male	Radiocephalic	Yes	Yes	Yes	No	No	Diabetes mellitus

Technical success was achieved in all 10 (100%) fistulas. Fistulograms obtained after sequential balloon maturation demonstrated a smooth luminal wall, an increase in venous caliber, and an absence of contrast extravasation, both at the treated segment and along the entire usable length of the fistula. No venous rupture, access-site hematoma, or post-procedural thrombosis was observed. Every fistula was subsequently cannulated with two needles and supported a complete hemodialysis session, giving a clinical success rate of 100%; no patient in this series required a repeat BAM session. Patients were followed clinically until successful functional use of the fistula for maintenance hemodialysis. Long-term primary, assisted primary, and secondary patency were not evaluated because follow-up beyond establishment of functional dialysis access was outside the scope of this case series. Procedural details and outcomes are summarized in Table 3.

**Table 3:** Procedural characteristics and outcomes of balloon-assisted maturation. Technical success was defined as successful crossing and dilatation of the target lesion with restoration of satisfactory angiographic flow, improvement in luminal caliber, and absence of significant residual stenosis or contrast extravasation on the completion angiogram, as assessed by the operating interventional cardiologist. BAM = balloon-assisted maturation

Variable	Value
Total patients/Fistulas treated	10/10
Mean age (range)	60.7 years (46-73)
Male:female	8:2
Radiocephalic fistulas	8 (80%)
Brachiocephalic fistulas	2 (20%)
Anesthesia	Local infiltration anesthesia
Imaging guidance	Fluoroscopy with iodinated contrast fistulography
Vascular access route	Antegrade puncture of the outflow (cephalic) vein
Lesion crossing	Hydrophilic 0.035-inch guidewire
Balloon angioplasty	Sequential dilatation of the juxta-anastomotic and outflow venous segments, sized to the adjacent non-stenotic vein
Technical success	10/10 (100%)
Clinical success (two-needle cannulation achieved)	10/10 (100%)
Contrast extravasation or venous rupture	0 (0%)
Access-site hematoma	0 (0%)
Post-procedure fistula thrombosis	0 (0%)
Additional (repeat) BAM required	0 (0%)

**Figure 3:**
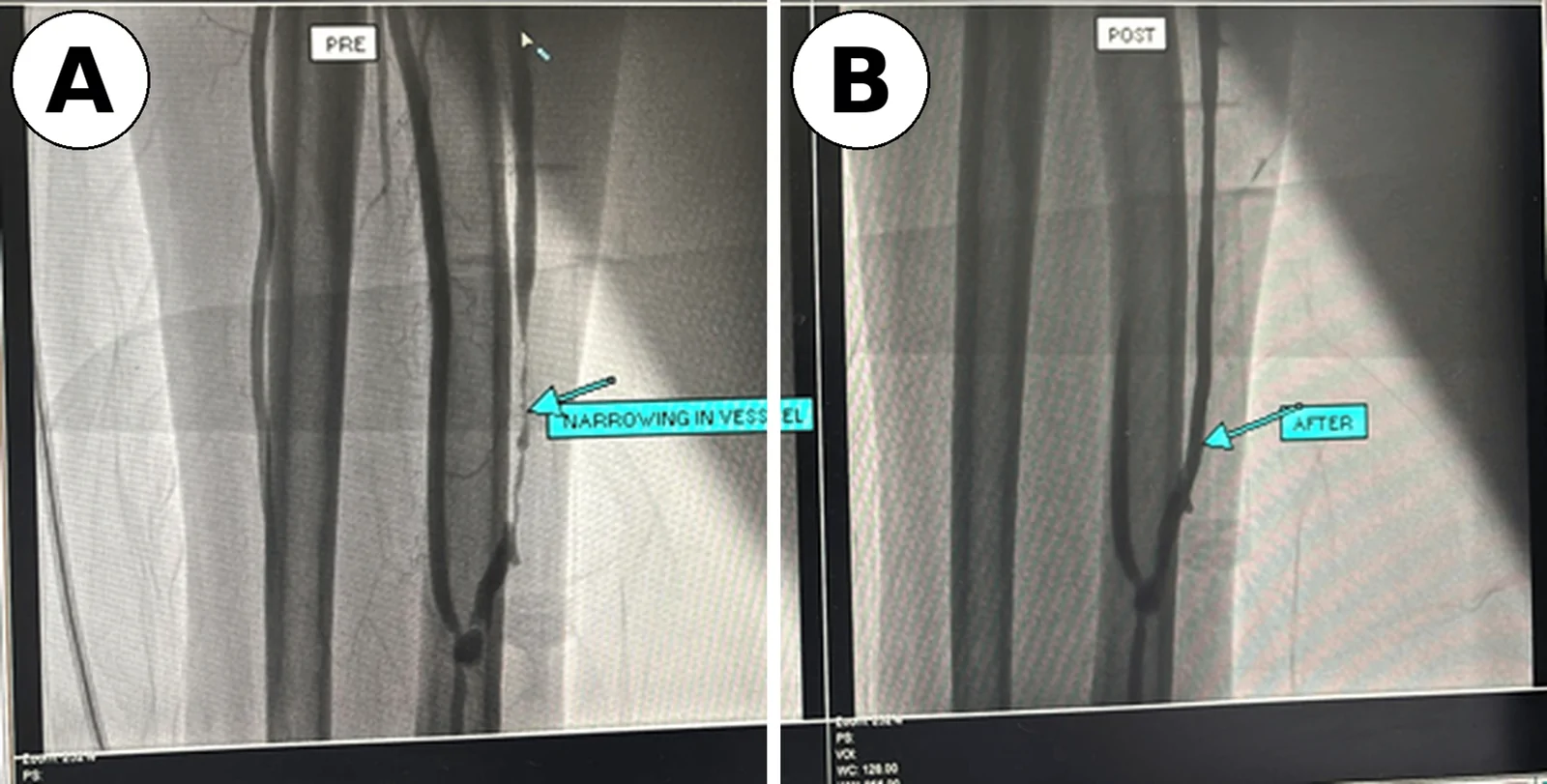
Left radiocephalic fistula before and after balloon-assisted maturation. (A) Pre-procedure fistulogram showing a stenotic segment of the outflow vein. (B) Post-procedure fistulogram showing increased luminal caliber and a smooth vessel wall after sequential balloon dilatation.

**Figure 4:**
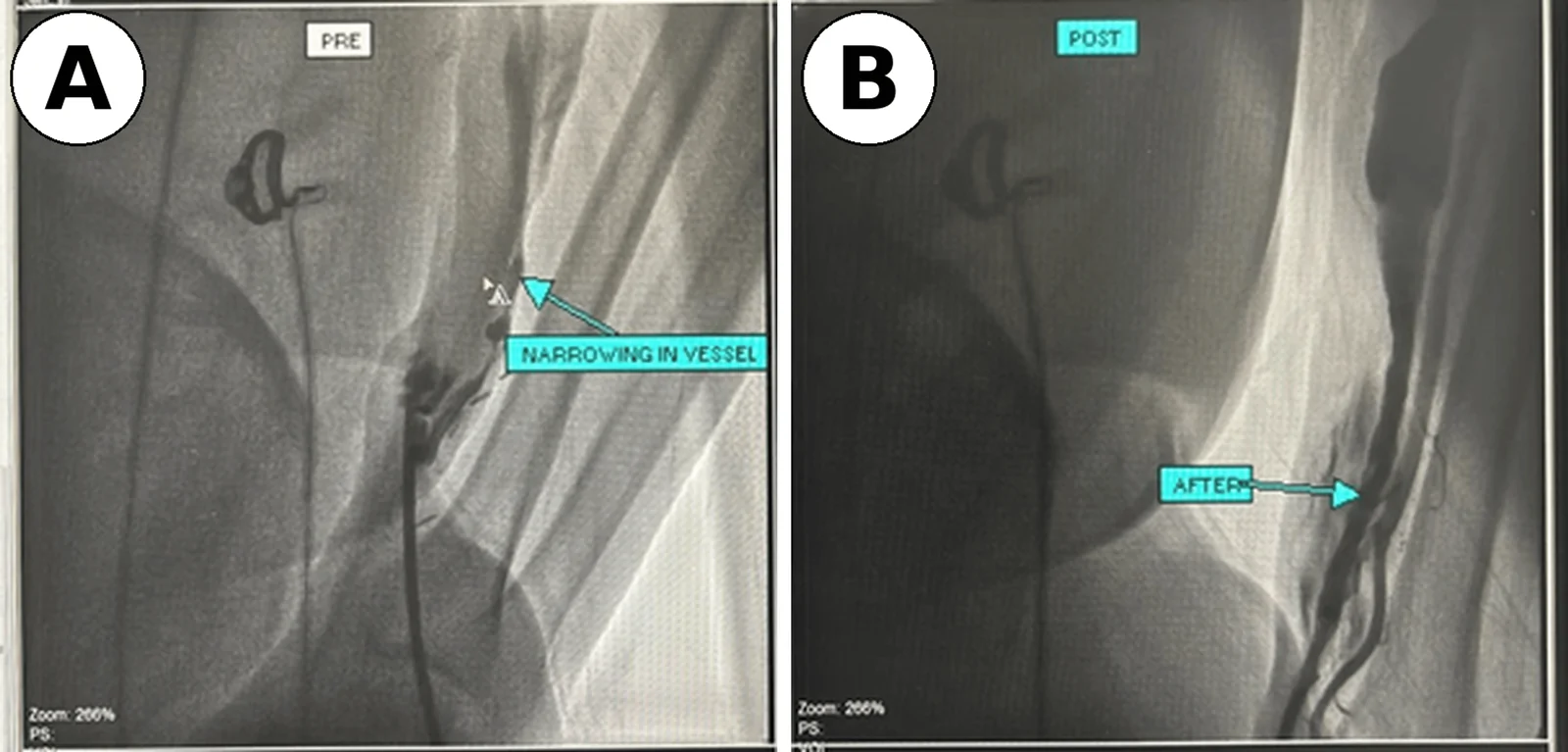
Left brachiocephalic fistula before and after balloon-assisted maturation. (A) Pre-procedure fistulogram showing stenosis of the outflow vein. (B) Post-procedure fistulogram showing successful dilatation with restoration of luminal patency.

**Figure 5:**
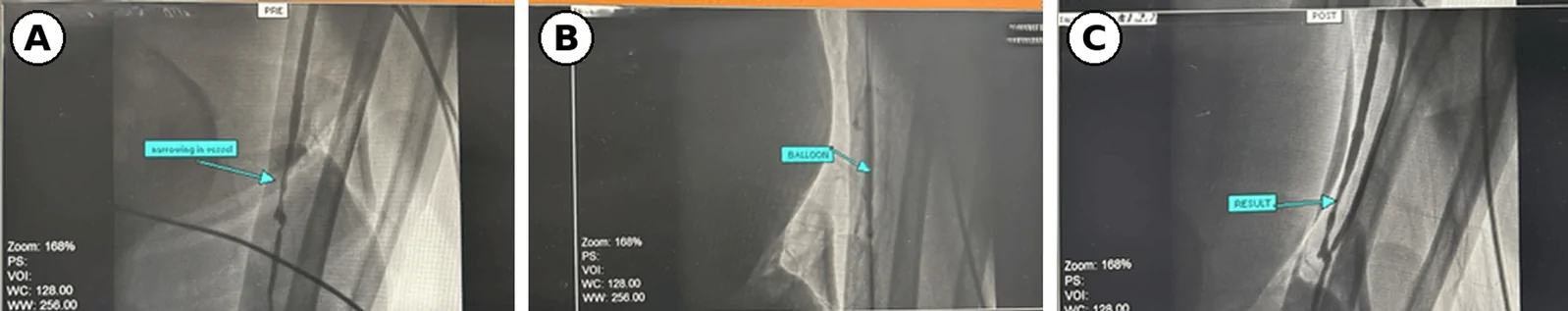
Left radiocephalic fistula before, during, and after balloon-assisted maturation. (A) Pre-procedure fistulogram showing narrowing of the vessel. (B) Fluoroscopic image during balloon angioplasty at the stenotic site. (C) Post-procedure fistulogram showing improved luminal diameter following dilatation.

The angiographic appearance of these lesions and their response to BAM are illustrated in the accompanying fistulograms. In each case, the pre-procedure image demonstrated a discrete stenosis with reduced luminal caliber at the juxta-anastomotic or outflow venous segment; the post-procedure fistulogram in each case showed resolution of the stenosis, an increase in venous caliber, and a smooth luminal wall, consistent with the angiographic endpoint used to define technical success in this series. The representative fistulograms from four of the treated patients are shown in Figures 3-5.

## Discussion

In this small consecutive series, BAM salvaged all 10 stenosed, non-maturing fistulas, with no procedural complications and with every fistula proceeding to two-needle cannulation. At a radiological level, maturation failure is typically characterized by stenosis of the peri-anastomotic venous segment; at a biological level, it reflects aggressive venous neointimal hyperplasia in which myofibroblast migration, inflammatory signalling, and oxidative stress prevent the adaptive outward remodelling required to convert a thin-walled vein into a cannulable conduit [4]. BAM addresses this mechanically, and by dilating the whole usable length of the fistula rather than a single focal lesion, it promotes both luminal gain and progressive remodelling.

Our success rate should be interpreted with caution, as it is derived from only 10 patients. It is nonetheless consistent with the upper end of the published range. The pooled meta-analytic estimates of 97% technical and 90% clinical success provide the most reliable benchmark [7]. Single-center series report more variable figures: Li et al. reported an overall success rate of 77.7% among 148 patients, with no statistically significant difference between radiocephalic (81.2%) and brachiocephalic (64.5%) fistulas, although the pattern of culprit lesions differed, with juxta-anastomotic and arterial inflow lesions predominating in radiocephalic fistulas and outflow lesions in brachiocephalic fistulas [9]. Kohiyama et al., using a transradial approach in 205 patients, achieved 100% technical success but a clinical success rate of 78%, with 45 patients requiring further procedures to achieve maturation [10]. A comparison of the present series with contemporary published cohorts is provided in Table 4.

**Table 4:** Comparison of the present series with contemporary published studies of balloon-assisted maturation. Studies differ in design, definitions of success, and duration of follow-up; the comparison is therefore descriptive and should not be interpreted as a formal pooled analysis. BAM = balloon-assisted maturation; AVF = atrioventricular fistula

Study	Design	Fistulas/Patients (n)	Reported success	Key observation
Vignesh et al. [8]	Single-center retrospective	23 patients (27 salvage procedures)	Technical success 88.8%; clinical success 81.5%	Endovascular salvage with acceptable mid-term patency
Kanchanasuttirak et al. [7]	Systematic review and meta-analysis	1,427	Clinical success 90%; technical success 97%	Complications reported in 1.7–41%; earlier BAM associated with better clinical success
Li et al. [9]	Single-center retrospective	148	Overall success 77.7%	No significant difference between radiocephalic (81.2%) and brachiocephalic (64.5%) fistulas
Kao et al. [11]	Single-center retrospective	61	36 of 38 salvaged fistulas matured	Acceptable long-term patency even with veins ≤3 mm
Huang et al. [12]	Single-center retrospective	290	Secondary maturation 94.1% vs. 90.3%	BAM equalized maturation between small (2.5–3 mm) and normal (≥3 mm) veins
Wang et al. [13]	Single-center retrospective	596	Overall maturation 83.1%	Small veins (<2.5 mm) achieved 75.9% maturation with BAM; 91% needed only one or two sessions
Kohiyama et al. [10]	Single-center retrospective	205	Technical success 100%; clinical success 78%	Transradial access; no access-site complications
Juneja et al. [14]	Single-center retrospective	239	Maturation 97% (≥7 mm balloons) vs. 88% (<7 mm)	Larger balloons associated with fewer re-interventions and faster catheter-free dialysis
Bae et al. [15]	Single-center retrospective	23	Maturation 87%	Staged full-length balloon angioplasty; no rupture or hematoma
Present series	Prospective case series	10	Technical and clinical success 100%	No procedural complications; all fistulas cannulated with two needles

An important consequence of BAM is that it widens the anatomical criteria for fistula creation. Traditional teaching held that veins smaller than 3 mm should be abandoned. Wang et al. showed that veins measuring less than 2.5 mm could still achieve a 75.9% maturation rate when BAM was used, with 91% of patients requiring only one or two sessions [13]. Huang et al. found that although the primary maturation rate was lower for veins of 2.5-3 mm than for larger veins, the secondary maturation rate after BAM was equivalent in the two groups (94.1% versus 90.3%) [12]. Kao et al. reported acceptable long-term functional patency after BAM even in radiocephalic fistulas created with veins of 3 mm or less, and identified the number of BAM procedures required as an independent predictor of primary functional patency [11]. Comprehensive preoperative ultrasound mapping, combined with the selective availability of BAM, has been shown to permit satisfactory maturation in veins as small as 1.35 mm after tourniquet augmentation [16]. In the present series, both brachiocephalic and radiocephalic fistulas were salvaged, which is in keeping with the observation that BAM reduces the need to move to a more proximal access simply because the distal vessels are small.

Several technical refinements of BAM have been described. Voto and Panetta performed primary balloon angioplasty at the time of fistula creation followed by staged maturation through a 4-French radial system, and achieved a fistula creation rate of 90.8% in a cohort with a high prevalence of diabetes and suboptimal veins [17]. Juneja et al. found that when the fistula tolerated balloons of 7 mm or more at the initial session, the maturation rate was higher (97% versus 88%), fewer re-interventions were needed, and the time to catheter-free dialysis was shorter [14]. Bae et al. used staged, serially upsized full-length balloon catheters approximately two weeks apart to minimize overlap-related barotrauma to the immature vein, and reported an 87% maturation rate with no ruptures or hematomas [15]. Alternative access routes have also been explored: an antegrade radial pull-through technique without sheath insertion produced success and patency rates comparable with the conventional venous approach [18], and a snuffbox distal transradial approach for fistuloplasty has been reported as safe and effective [19]. Our practice of accessing the outflow vein and pre-dilating the juxta-anastomotic segment before sequential dilatation sits within this spectrum of accepted techniques.

Whether drug-coated balloons offer an advantage over plain balloons in this setting remains unsettled. Mirabella et al. used intraoperative Doppler-guided BAM with drug-eluting balloons at the time of first fistula creation and reported successful maturation without secondary procedures [20]. At the level of pooled evidence, however, the picture is inconsistent: one meta-analysis of randomized trials found significantly higher target-lesion primary patency with drug-coated balloons at both six and 12 months [21], while an earlier meta-analysis found no significant patency benefit at either time point [22]. Plain balloon angioplasty therefore remains a reasonable default, and drug-coated devices cannot yet be regarded as standard of care for maturation. Where angioplasty alone fails, adjunctive strategies such as interwoven nitinol stent-assisted maturation [23] or surgical elevation of an excessively deep fistula [24] may be considered.

Fistula maturation ultimately requires a compliant, responsive vasculature capable of dilating in response to the increased flow velocity of the newly created low-resistance circuit, and this process is usually complete within the first weeks after creation. Failure to achieve maturation within four to eight weeks should prompt a search for a reversible cause rather than expectant observation, as prolonged reliance on a tunnelled catheter carries its own burden of infection and central venous stenosis [1]. Our protocol of structured review at four and eight weeks with early referral for angioplasty is aligned with this principle, and with the meta-analytic observation that earlier BAM is associated with better clinical success [7]. Although limited by sample size, this study contributes one of the few contemporary Indian case series describing balloon-assisted maturation of non-maturing autogenous fistulas. The findings demonstrate that timely endovascular intervention can be incorporated into routine vascular access practice with excellent immediate technical success and may reduce prolonged catheter dependence in appropriately selected patients.

Limitations

This study represents the early institutional experience with BAM and therefore includes only 10 consecutive patients treated during the study period. Although the sample size is small and there was no comparator group, consecutive inclusion of all eligible patients minimizes selection bias and reflects real-world clinical practice. Consequently, no inference can be drawn regarding the comparative efficacy of BAM versus conservative management, surgical revision, or creation of a new vascular access. Follow-up was limited to the period required to establish successful cannulation, and formal primary, assisted primary, and secondary patency rates were not calculated; the durability of the result therefore remains unknown. Time to maturation was not measured systematically, and we are consequently unable to substantiate the impression that BAM shortens the maturation interval. Detailed procedural variables including vessel diameter, balloon diameter, and inflation pressure were not recorded uniformly for all patients, limiting technical reproducibility. Detailed procedural variables, including vessel diameter, balloon diameter, and inflation pressure, were not recorded uniformly for all patients, limiting technical reproducibility. Finally, the 100% technical and clinical success observed in this series should be interpreted cautiously given the small sample size and the inherent limitations of a single-center case series, and should not be considered representative of outcomes in all clinical settings.

## Conclusions

In this consecutive series of 10 patients with stenosed, non-maturing AVFs, BAM was performed safely under local anesthesia and achieved technical and clinical success in every case, with no procedural complications and with all fistulas subsequently cannulated for hemodialysis. These findings are consistent with the larger published literature, in which BAM reliably salvages non-matured fistulas and extends the range of veins that can be used for autogenous access. The small sample, the absence of a control group, and the lack of long-term patency data mean that our results should be regarded as hypothesis-generating. Adequately powered prospective studies with predefined patency endpoints, systematic recording of vessel and balloon dimensions, and longer follow-up are needed to define the place of BAM within the end-stage kidney disease access life-plan, and to clarify which patients benefit most. Our experience also supports early surveillance and timely endovascular intervention as practical strategies for maximizing successful autogenous fistula utilization in routine clinical practice.
